# Neighborhood characteristics and psychological distress of long-term immigrants in California

**DOI:** 10.1186/s12889-025-25766-7

**Published:** 2025-11-26

**Authors:** Jan A. Wagstaff, Janice F. Bell, Marta Induni, Leigh Ann Simmons, Victoria F. Keeton

**Affiliations:** 1 Betty Irene Moore School of Nursing, University of California, Davis, 2570 48th St, Sacramento, CA 95817 USA; 2 Public Health Institute Center for Immigrant and Refugee Health, 1750 Howe Ave #550, Sacramento, CA 95825 USA

**Keywords:** Immigrant, Psychological distress, Neighborhood, Community, Mental health, CHIS, Acculturation

## Abstract

**Background:**

Immigrants and their communities face unique challenges that exacerbate their risk for psychological distress. For long-term immigrants, these challenges may intensify over time, leading to feelings of isolation, frustration, and marginalization, contributing to higher levels of stress. Neighborhood programs that support community-building and connection may be an effective solution, however, evidence to inform such interventions is sparse. The aim of this study was to examine associations between neighborhood characteristics and the levels of psychological distress among long-term California immigrants.

**Methods:**

Using data from the biannual 2021-2022 California Health Interview Survey, we conducted a secondary analysis of responses from 4,595 immigrant participants who had lived in the U.S. for 15 years or longer. The primary outcome was psychological distress, as measured by scores on the Kessler 6 Psychological Distress Scale. We used Wilcoxon rank sum tests and separate simple linear regressions to test unadjusted associations between perceptions of four neighborhood-level characteristics (support, trust, safety, and getting along) and Kessler 6 scores. In a final multivariable linear regression model, we examined associations between all the neighborhood characteristics and Kessler 6 scores while controlling for relevant covariates.

**Results:**

In the final fully adjusted model, perceptions of neighborhood trustworthiness (β = -1.13; 95% CI: -1.69, -0.57; P < 0.001), safety (β = -1.62; 95% CI: -2.50, -0.74; P < 0.001), and getting along (β= -0.74; 95% CI: -1.46, -0.03; P < 0.001) were independently and significantly associated with lower psychological distress scores. Perceptions of neighborhood support did not have significant associations in the model.

**Conclusions:**

Characteristics of neighborhood trust, getting along, and safety, are positively associated with lower psychological distress in a sample of long-term California immigrants. Community-driven interventions promoting social connectedness and inclusivity among neighborhood residents while also supporting structures of safety and resource accessibility may promote and sustain well-being among immigrant populations.

## Background

California is home to the largest number of immigrants in the United States, with over a quarter of its residents born abroad [1]. Recent data demonstrated an alarming surge between 2015 and 2021 in psychological distress (PD) among immigrants residing in California, with those residing in the country for 15 years or more demonstrating a 50% increase in rates of distress [2]. This corroborates other evidence that the longer immigrants reside in their new country, the greater their psychological distress [3–6]. Reports of adverse mental health for immigrants contradict a phenomenon termed the *healthy immigrant effect*, derived from research showing lower rates of mental and physical illness among immigrants compared to native born individuals [7, 8]. The healthy immigrant effect has been challenged in part by evidence that immigrants with higher levels of acculturation have more adverse health outcomes [9, 10], suggesting that greater acculturation and/or duration of time spent in the foreign country may play an important role. While immigrants increasingly experience distress in the short-term [2], especially related to socioeconomic and structural challenges related to migration, settlement, and integration [11], consistent evidence demonstrates that the mental health of immigrants declines over time – this has been termed the *years since immigration effect* [7]. The acculturation process for immigrants is often associated with growth and enrichment and generally perceived to be a positive experience [12], however, its effects on health appear to be more nuanced and complex [7].

Some risk factors for psychological distress among immigrants are similar to those experienced by the general population, such as financial hardships, identifying as female, and having poor health [13]. However, immigrants also face unique challenges that exacerbate their risk, and these challenges tend to intensify over time. Structural inequities in access to education, healthcare, and citizenship present persistent barriers, creating ongoing uncertainty and anxiety [14]. For long-term residents, this difficulty can lead to feelings of isolation, frustration, and marginalization, contributing to higher levels of stress [15]. Prolonged exposure to economic and legal strains, such as supporting families, navigating immigration systems, or sending remittances abroad, only adds to the psychological burden [16–18]. Financial problems also can be persistent for foreign-born older adults who earn less over their lifetimes and accumulate less wealth than those born in the U.S [19]. Furthermore, as immigrants spend more time in the U.S., they may face growing tension between maintaining their cultural identity and adapting to the expectations of a new society, leading to acculturation stress [20, 21]. Intergenerational conflicts can emerge within families around values, resulting in conflict, alienation, and isolation [20, 22, 23]. In the current political climate, reports of Immigration and Customs Enforcement (ICE) detaining not only undocumented people but legal residents and citizens, has deepened mistrust and heightened collective anxiety within immigrant communities [24].

Given that some of the stressors affecting immigrants over time involve exacerbated isolation and marginalization, a logical avenue for intervention is to examine structures and policies that support community-building and connection. Indeed, studies of recent immigrants suggest that community-based interventions can profoundly, positively impact their life and health trajectories [25]. However, funding and resources to support immigrants in the U.S. primarily target recent arrivals, offering supports such as language training and job assistance [26]. Further, the specific role that neighborhood characteristics—such as community support networks, availability of amenities, or exposure to crime—play in either alleviating or exacerbating psychological distress for long term immigrants remains underexplored. These gaps highlight the need for focused studies that inform and evaluate community-level characteristics associated with the well-being of long-term immigrants. To this end, the aim of this study was to examine associations between neighborhood characteristics—such as the willingness of neighbors to help one another, overall neighbor relations, trust, and perceptions of neighborhood safety—and the levels of psychological distress among California immigrants who have resided in the U.S. for 15 years or more.

## Methods

### Study design and setting

This study is a secondary analysis of publicly-available data from the 2021–2022 California Health Interview Survey (CHIS), conducted by the University of California, Los Angeles, Center for Health Policy Research [27]. CHIS is a biennial, population-based, cross-sectional survey with ongoing data collection over two-year cycles [28]. CHIS is used to gather comprehensive health information from a representative sample of California residents recruited through an address-based sampling method, which enables them to reach respondents who may not have landline telephones. Geographical stratification and oversampling in some regions ensures representation across regions and demographic characteristics such as age, gender, race, ethnicity, and income. One adult from each household is selected for an interview and acknowledges consent for participation after their eligibility is confirmed and before the interview begins [28]. CHIS has gone through extensive translation and cultural adaptation processes [27, 29] and interviews were conducted in nine languages: English, Spanish, Cantonese, Vietnamese, Tagalog, Mandarin, Korean, Asian Indian, and Russian.

### Participants

The 2021–2022 CHIS survey included a total of 21,452 adult respondents, with 5,597 (31%) identifying as immigrants and 15,866 (69%) born in the U.S. The sample used for analysis in this study was limited to respondents with non-missing data who were born outside the U.S. (*N* = 5,595) and had lived in the U.S. for 15 years or more, for a final sample of *N* = 4,595 participants. Due to the analysis of publicly available, de-identified data, the study was deemed exempt by the University of California, Davis Institutional Review Board.

### Measures

#### Psychological distress

The 2021–2022 CHIS included questions from the widely used Kessler 6 Psychological Distress Scale (Kessler 6) [30, 31], which has been validated across different populations and in numerous languages [32]. The Kessler 6 instrument instructs respondents to think about one month in the past 12 months when they were at their worst emotionally. They are then asked to rate how often in that month they had each of the following six feelings: *nervousness*,* hopelessness*,* worthlessness*,* restlessness or fidgetiness*, finding *nothing could cheer them up*, and finding *everything was an effort*. Response options were scored on a five-point Likert scale of *all (4)*,* most (3)*,* some (2)*,* a little (1) of the time or not at all (0)*. The numerical values for each response were summed to produce a PD score ranging from 0 to 24. The primary outcome for this study was the total PD score for the worst month in the previous 12 months, analyzed as a continuous variable.

#### Neighborhood characteristics

We examined four independent variables measuring participants’ perceptions of their neighborhoods using questions from the CHIS. Three were adapted from the Project on Human Development in Chicago Neighborhoods (PHDCN) Community Involvement and Collective Efficacy questionnaire and the social cohesion scale [33, 34]. The fourth was an additional question about safety. The PHDCN has been widely used in research for over two decades in English and Spanish [33, 35]. Respondents were asked to rate their level of agreement with the following statements: “*People in my neighborhood are willing to help each other*” (support); “*People in this neighborhood generally* *do not* *get along with each other*” (get along); and “*People in this neighborhood can be trusted*” (trust). These were rated on a four-point Likert scale from *strongly agree* to *strongly disagree* and we recoded them as dichotomous (agree/strongly agree vs. disagree/strongly disagree). In all regression analyses, the support and trust variables were coded with disagree/strongly disagree as the reference group while getting along was reverse coded with agree/strongly agree as the reference group. The fourth neighborhood variable was measured by responses to the question, “*Do you feel safe in your neighborhood?*” (safety), using a four-point Likert scale from *all of the time* to *none of the time* (recoded as dichotomous to some/none of the time = reference, most/all of the time).

#### Covariates

We included additional covariates in the adjusted regression model for their known or theoretical influences on either the outcome or independent variables, collapsing categories with lower cell counts as needed for analysis: gender [36, 37] (male = reference, female), race and ethnicity [38] according to the University of California, Los Angeles Center for Health Policy Research definitions (recoded as Asian = reference; Latino; other single/multiple race/African American/American Indian/Alaska Native; White), age in years [39–41] (recoded as 18 to 29 = reference, 30 to 49, 50 to 64, 65 and older), English language proficiency [42] (recoded as speaks English well/only = reference, does not speak English well/at all), citizenship status [43–45] (naturalized = reference, non-citizen), highest level of education [46–48] (recoded as higher than bachelor’s degree = reference, bachelor’s degree, high school diploma/vocational school/associate’s degree/some college, less than high school diploma), annual income percentage of the federal poverty level [44, 47] (300% and higher = reference, 200–299%, 100–199%, 0–99%), percentage of life spent living in the U.S [45, 49] (recoded as 0–40%=reference, 41–60%, 61–80%, 81% and higher), years lived at current address (recoded as 0–4 years = reference, 5–14 years, 15 years and higher), and self-rated general health [49–51] (recoded as good/very good/excellent = reference, fair/poor). We included both the variables for age and percentage of life spent living in the U.S. to account for potential long-term effects of differences in age at the time of immigration in addition to general effects of age on mental health [52]. We hypothesized this could provide a more nuanced control for how both age and acculturation influence psychological distress among long-term immigrants. We accounted for potential overlap between these variables in our analyses, as described below.

### Data analysis

Data were analyzed with Stata statistical software, version 18.5 (StataCorp, 2024. College Station, TX). The outcome, primary independent variables, and sociodemographic and health characteristics of the sample were summarized with descriptive statistics. Median PD scores for each of the four neighborhood characteristic variables were compared using Wilcoxon rank sum tests to account for their positively skewed distributions. Following initial comparisons using Wilcoxon tests, regression analyses were conducted to examine associations. We used separate simple linear regression models to test unadjusted associations between the PD score and each of the independent variables and covariates. A final single multivariable linear regression model was used to examine associations between PD score and all of the neighborhood characteristic variables controlled for all covariates. Prior to conducting survey-weighted analyses, we assessed multicollinearity among independent variables and covariates. Variance inflation factors were generally low (mean = 2.62), and only age categories showed higher values (8–11). In comparing the model with and without the age variable, our examination of coefficient stability and standard errors indicated that including age did not significantly affect other estimates, so the final model retained all covariates. In the final model, the sampling weights provided by CHIS were applied to account for the complex sampling design and yield estimates generalizable to the non-institutionalized, California population [28].

## Results

### Sample characteristics

Table 1 summarizes the sociodemographic, health, and neighborhood characteristics of the sample. Over two-thirds (70.4%) were between 30 and 64 years of age and almost one-quarter (24.2%) were older than 64 years. There was a relatively even gender mix with 51.5% identifying as female. The largest racial/ethnic groups were Latino (40.2%) and Asian (28%). There were 19.1% identifying with multiple race/ethnic identities and 12.5% White. Educational levels were fairly evenly distributed with just over a third reporting less than a high school diploma (35.8%), almost a third having a high school diploma, vocational education or some college (32.2%), and just under a third holding a Bachelor’s (18.2%) or higher (13.6%) degree. Nearly half of the sample reported a household income of 300% above the federal poverty level. English was the only language spoken or spoken with proficiency in a majority of households (73.3%), and most (71.6%) were naturalized citizens. Over half of the sample (52.7%) had spent more than 60% of their lives in the U.S., and more than two-thirds (69.2%) had lived at their current address for over five years. Three-quarters of the sample (75.2%) reported very good or excellent health, and the median Kessler-6 score for PD was 3 out of 24 (interquartile range [IQR]: 1, 8).

**Table 1:** Characteristics of immigrants living in the U.S. for 15 years or longer (N = 4595)

	N	weighted %
Age group (years)		
18 to 29	118	5.35
30 to 49	1223	34.57
50 to 64	1877	35.83
65+	1377	24.25
Self-identified gender		
Female	2463	51.56
Male	2132	48.44
Self-identified race/ethnicity		
Asian	1804	28.05
Latino	1294	40.23
Other/multi/AA/AI/AN	783	19.19
White	714	12.53
Highest education		
Higher degree	1004	13.64
Bachelor's degree	1266	18.29
High school/vocational/some college	1648	32.27
Less than high school	677	35.80
Household income % of federal poverty level		
300% or higher	2513	45.40
200-299%	550	13.21
100-199%	825	21.62
0-99%	707	19.77
English Proficiency		
Speaks English only or well	3684	73.34
Does not speak English well or at all	911	26.66
Citizenship status		
Naturalized citizen	3699	71.68
Non-citizen	896	28.32
Percent of life spent in the U.S.		
0-40%	543	10.93
41-60%	1488	36.30
61-80%	1484	33.27
81+%	1080	19.50
Time lived at current address		
0-4 years	1426	30.72
5-14 years	1524	34.51
15+ years	1645	34.77
General Health Condition		
Very good/excellent	3574	75.22
Fair/poor	1021	24.78
Kessler-6 Score in Worst of Last 12 Months (median, IQR)		
Psychological Distress	3	(1, 8)
Neighbors willing to help each other	3767	80.70
Neighbors can be trusted	3742	78.30
Feel safe in neighborhood	3975	83.88
Neighbors get along	3763	81.45

*AA* African American, *AN* Alaska Native, *AI* American Indian

**Fig. 1:**
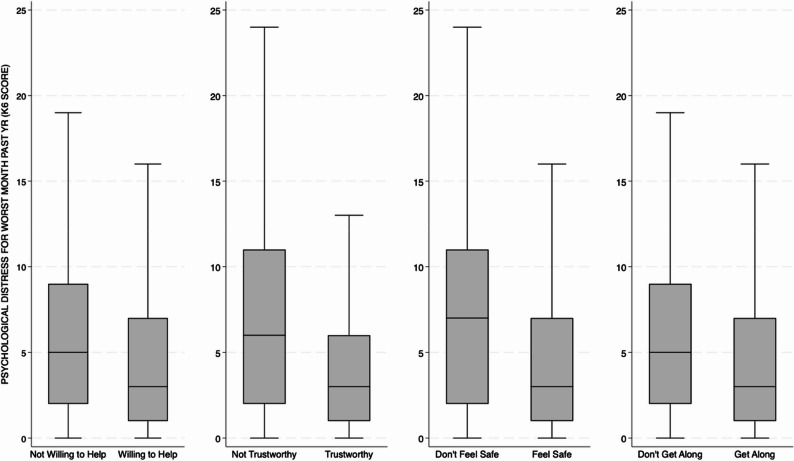
Median psychological distress scores comparisons by neighborhood characteristics*. *All comparisons significant at P < 0.001

The majority of the sample perceived their neighbors as willing to help one another (80.7%) and trustworthy (78.3%), and disagreed with the statement that their neighbors do not get along (81.4%). Most also reported feeling safe in their neighborhoods (83.8%). Participants who reported favorable perceptions of neighborhood characteristics had significantly lower median PD scores (*P* < 0.001) when compared to those with unfavorable perceptions (Fig. 1).

### Primary outcome

Table 2 presents the unadjusted linear regression models of PD scores upon each of the neighborhood characteristics. In each model, positive neighborhood characteristics were significantly associated with lower psychological distress. Perception of neighbors’ support for each other was associated with a 1.79 point decrease in PD score (95% confidence interval [CI]: −2.37, −1.21; *P* < 0.001), and trustworthiness was associated with a 2.34 point decrease (95% CI: −2.83, −1.85; *P* < 0.001), when compared to participants who did not report agreement with those characteristics. Those who reported feeling safe in their neighborhood most or all of the time had the strongest negative association, with − 2.82 points lower PD scores than those reporting feeling safe some or none of the time (95% CI: −3.63, −2.01; *P* < 0.001). PD scores were also − 1.55 points lower for participants who disagreed with the perception that their neighbors did not get along when compared with those who agreed (95% CI: −2.16, −0.94; *P* < 0.001).

**Table 2:** Unadjusted linear regressions of serious psychological distress on neighborhood solidarity characteristics (*N* = 4595)

	β	(95% CI)			*P*
Neighbors willing to help each other (disagree = ref)	−1.79	−2.37		−1.21	< 0.001
Neighbors can be trusted (disagree = ref)	−2.34	−2.83		−1.85	< 0.001
Feel safe in neighborhood (some/none = ref)	−2.82	−3.63		−2.01	< 0.001
Neighbors do not get along (agree = ref)	−1.55	−2.16		−0.94	< 0.001

Results reported are for separate models of the outcome on each independent variable with survey weights applied, without adjusting for covariates

*ref* Reference, *CI* Confidence interval, *P* level of significance

In the final model including all neighborhood characteristics together and adjusting for covariates, trustworthiness (β = −1.13; 95% CI: −1.69, −0.57; *P* < 0.001), safety (β = −1.62; 95% CI: −2.50, −0.74; *P* < 0.001) and getting along (β = −0.74; 95% CI: −1.46, −0.03; *P* < 0.001; Table 3) were independently and significantly associated with lower PD scores. Perceptions of neighbors’ support for each other did not have significant associations with PD scores in this model. Covariates that were significantly associated with lower PD scores included older age and greater time lived at current address.

**Table 3:** Adjusted multiple linear regression of serious psychological distress on neighborhood characteristics (N = 4595)

	β		(95% CI)		*P*	
Neighbors willing to help each other (disagree=ref)	-0.35	-0.98		0.27	0.26	
Neighbors can be trusted (disagree=ref)	-1.13	-1.69		-0.57	< 0.001	*
Feel safe in neighborhood (some/none=ref)	-1.62	-2.50		-0.74	< 0.001	*
Neighbors do not get along (agree=ref)	-0.74	-1.46		-0.03	0.04	*
Age Group (years)						
18 to 29 (ref)	---	---		---	---	
30 to 49	-3.17	-4.56		-1.78	< 0.001	*
50 to 64	-4.53	-6.00		-3.07	< 0.001	*
65+	-5.03	-6.57		-3.50	< 0.001	*
Self-Identified Gender						
Male (ref)	---	---		---	---	
Female	0.90	0.45		1.35	< 0.001	*
Self-identified race/ethnicity						
Asian (ref)	---	---		---	---	
Latino	-0.00	-0.61		0.61	0.99	
Other/multi/AA/AI/AN	0.15	-0.45		0.76	0.60	
White	0.69	0.13		1.26	0.01	*
Highest Education						
Higher degree (ref)	---	---		---	---	
Bachelor's degree	-0.02	-0.69		0.63	0.93	
High school/vocational/some college	-0.50	-1.05		0.05	0.07	
Less than high school	-0.67	-1.52		0.18	0.12	
Household Income % of Federal Poverty Level						
300% or higher (ref)	---	---		---	---	
200-299%	0.66	0.01		1.30	0.04	
100-199%	0.68	0.09		1.27	0.02	
0-99%	0.93	0.21		1.66	0.01	*
English Proficiency						
Speaks English only or well (ref)	---	---		---	---	
Does not speak English well or at all	-0.46	-1.02		0.09	0.10	
Citizenship status						
Naturalized citizen (ref)	---	---		---	---	
Non-citizen	-0.56	-1.18		0.05	0.07	
Percent of Life Spent in the U.S.						
0-40% (ref)	---	---		---	---	
41-60%	0.09	-0.52		0.72	0.75	
61-80%	0.55	-0.21		1.31	0.15	
81+%	0.99	0.25		1.74	0.009	*
Time lived at current address						
0-4 years (ref)	---	---		---	---	
5-14 years	-0.94	-1.52		-0.37	0.002	*
15+ years	-0.82	-1.47		-0.17	0.01	*
General Health Condition						
Very good/excellent (ref)	---	---		---	---	
Fair/poor	2.37	1.74		2.99	< 0.001	*

*ref* Reference, *CI* Confidence interval, *AA* African American, *AN* Alaska Native, *AI* American Indian

*denotes significance at P < 0.05

## Discussion

In this study, neighborhood characteristics–specifically perceptions of trust, getting along, and safety–were associated with lower PD scores among CHIS respondents who immigrated to the U.S. and have been in the country for 15 years or longer. This supports prior research that social connections and strong community ties contribute to well-being for immigrants in general [53–57], and extends it to highlight the potential role of neighborhood characteristics as protective against the risks for psychological distress in long-term immigrant residents. Recent literature promotes neighborhood-level interventions to advance health equity [58], and these findings inform ways in which such efforts can be shaped to address mental health disparities for immigrants [7]. Protective factors, such as community connections and social integration, support resilience, a critical factor in the well-being of immigrants as the length of residency increases [59].

As their time spent living in the country increases, long-term immigrant residents face unique stressors related to cultural integration, discrimination, and shifting neighborhood conditions, which may affect their mental health [39]. In mixed-status households, where some family members are long term citizens or lawful residents and others are undocumented, chronic stresses from legal insecurity, ongoing fears of deportation, social exclusion, family separation, and limited access to resources can be exacerbated [43, 60]. Families and individuals may be reluctant to participate in community engagement, which undermines the protective effects of neighborhood trust and getting along, even in otherwise supportive environments [43, 60]. Communities and neighborhoods may struggle to welcome immigrant residents and view their presence unfavorably [25, 61]. This negative perception from local residents can create an environment of exclusion and hostility, making it challenging for immigrants to integrate and thrive [25, 39, 61]. Lack of social support and diminished sense of community contribute to heightened feelings of isolation and stress [53, 62]. Community-based interventions aimed at addressing these challenges build greater cohesion and trust within neighborhoods, thereby promoting positive mental health among immigrant residents [25, 63]. Although we were specifically interested in the long-term immigrant population, neighborhood interventions could improve well-being for *all* immigrants. Future research should examine neighborhood influences on short-term immigrants to better understand how initial adaptation, legal precarity, and social integration intersect with community characteristics.

Neighborhood safety had the strongest association with low distress in our sample, in line with previous findings that neighborhood crime is a significant predictor of mental health outcomes [64]. The concentration of immigrants in city neighborhoods can cause local residents from majority racial or ethnic groups to relocate in search of more homogenous communities, and this exacerbates socioeconomic differences and segregation within urban areas [61]. When this happens, amenities decline, access to resources and opportunities become limited, and these neighborhoods may experience a rise in crime and poverty [61, 65, 66]. High ethnic density within communities can lead to segregation, reducing interactions between diverse groups and weakening overall social cohesion and integration [53, 62]. Despite this, higher immigrant resident concentration is consistently associated with reduced crime [66, 67]. In one study, the number of immigrant-serving organizations moderated the effect of immigrant concentration on crime rates [67], suggesting that community resources may play an important role in supporting safety and subsequent mental health. Evidence also suggests that targeted efforts to increase neighborhood order and quality may build cohesion [68] and mitigate effects of socioeconomic deprivation on mental health in general [69, 70]. Community-driven initiatives that cultivate trust, encourage representation, and prioritize non-carceral responses may play an important role in strengthening safety, resilience, and social cohesion within vulnerable immigrant communities [24]. However, more research is needed to explore whether these effects hold true for immigrant-dense neighborhoods.

Neighborhood interventions have demonstrated positive outcomes for general populations [68, 69, 71], but gaps remain in understanding their role in fostering integration and enhancing well-being for immigrants. Prior research on neighborhood and mental health has focused on the built and natural environments, such as urbanicity, housing, and green spaces with less attention given to the social dynamics [72]. Our study addresses this gap by focusing on the social environment and its association with psychological distress. A recurring theme in successful neighborhood interventions, aligning with our findings on long-term immigrants, is the importance of addressing both structural conditions and social processes [68]. The links between mental health, interpersonal relationships, and social determinants of health are well documented [73].

Multilevel interventions are most likely to benefit long-term immigrant mental health [74], such as strategies that promote social connectedness and inclusivity among neighborhood residents while also supporting safety and accessible resources. For example, community navigators can act as intermediaries between individuals, the community, and public services, addressing multi-level influences on well-being [73]. Navigators engage hard-to-reach individuals, involving them in one-to-one support, education, group activities, and healthcare. Similarly, the use of lay health workers is an evidence-based multilevel intervention model where individuals connect with a worker who introduces them to community and structural services [75]. This has been shown to promote mental health and social equity [75].

Lastly, it would be remiss to discuss these findings without acknowledging the current sociopolitical environment that has immigrants across the U.S., regardless of naturalization or citizenship status, concerned about their futures and the futures of their families [1, 24]. Policy proposals ranging from the end of birthright citizenship to travel bans to restrictions on asylum access, which are compounded by significant increases in hate crimes based on race/ethnicity, will undoubtedly impact the mental well-being of this vulnerable group [76]. Future research and intervention development should be designed to ensure that resulting data can be used for evidence-based policymaking that may improve immigrant health equity [1].

### Limitations

Our findings should be considered within the context of five limitations. First, secondary analysis of data inherently limits the ability to explore potential confounding influences that were not collected in the primary study. For example, we did not have access to sufficient data on participants’ identified ethnic or sub-ethnic groups which precluded examination of whether our findings differed along these lines. We also lacked information on participants’ individual documentation status and whether they resided in mixed-status households, both of which may uniquely shape experiences of fear, exclusion, and psychological stress [43, 60]. Second, in an effort to maintain model parsimony and avoid overfitting, we limited the number of covariates in the analyses to the most pertinent to the study aims, and therefore may not have accounted for variables that also may influence psychological distress across populations, such as marital and family status or adverse childhood experiences, or affect neighborhood perceptions, such as property ownership status. Third, the sample did not include unhoused individuals or those living in institutionalized settings. Thus, results may not be generalizable to immigrants in those contexts, living in other states, or those who may not be comfortable participating in survey research. Fourth, three of the neighborhood variables we examined are part of a longer PHDCN social cohesion scale that was not fully included in CHIS, and we are not aware of any studies to validate the use of these individual items as independent constructs. Finally, CHIS applies rigorous translation techniques to ensure cross-cultural adaptation [29]; however, we did not find data to support that CHIS or the PHDCN have been psychometrically validated in English or other languages. These may affect internal validity and lead to systematic error, particularly with immigrants with lower English proficiency, and our findings - although not intended to infer causality - should nevertheless be interpreted with caution. Further research could better support the validity of these measures to strengthen their cross-cultural application.

## Conclusion

Positive neighborhood characteristics such as trust, getting along, and safety may play an important role in the psychological well-being of long-term immigrants in the U.S. Our findings highlight the importance of neighborhood-level interventions in supporting immigrant well-being and underscore the need for further research on the complex needs of long-term immigrant residents. As immigrant communities continue to face barriers to healthcare, education, and economic opportunities, the gap in well-being and access to essential services is likely to grow [77]. Neighborhood environments offer avenues to reduce disparities for long-term immigrants by addressing feelings of isolation and marginalization through social connection and resource provision in the places where they live. Immigrants contribute to the social, economic, and cultural fabric of our communities, yet many face persistent stressors that can undermine their well-being. Fostering inclusive, supportive neighborhoods not only benefits immigrant populations but also strengthens overall community resilience and cohesion [78].

## Data Availability

The dataset analysed during the current study is publicly available at the UCLA Center for Health Policy Research: https://healthpolicy.ucla.edu/our-work/california-health-interview-survey-chis/access-chis-data.

## Abbreviations

PD: Psychological distress
CHIS: California Health Interview Survey
Kessler 6: Kessler 6 Psychological Distress Scale
IQR: Interquartile range
CI: Confidence interval
